# Markers of Endothelial Injury and Dysfunction in Early- and Late-Onset Preeclampsia

**DOI:** 10.3390/life10100239

**Published:** 2020-10-14

**Authors:** Jakub Kornacki, Przemysław Wirstlein, Ewa Wender-Ozegowska

**Affiliations:** Division of Reproduction, Department of Obstetrics, Gynecology, and Gynecologic Oncology, Poznan University of Medical Sciences, 60-535 Poznan, Poland; abys@wp.pl (P.W.); ewaoz@post.pl (E.W.-O.)

**Keywords:** preeclampsia, endothelium, endothelial glycocalyx, cell adhesion molecules, endothelial dysfunction

## Abstract

With regard to differences in the clinical symptoms of preeclampsia (PE), the degree of endothelial dysfunction may differ between early and late-onset preeclampsia (EOP and LOP). The authors of this study examined it by assessing the endothelial injury level in women with EOP (20 patients) and LOP (20 patients) and in normotensive pregnant women (20 patients) in their late second and third trimesters of pregnancy, using the two markers—the serum concentration of hyaluronan (HA) and the serum level of soluble vascular cell adhesion molecule-1 (sVCAM-1). The serum concentrations of HA and sVCAM-1 did not differ significantly between the EOP and LOP patients. However, these were statistically higher than that of the control group participants (*p* < 0.05; *p* < 0.001). A significant correlation between the levels of HA and sVCAM-1 was found both in the entire group of patients with preeclampsia (*p* = 0.0277) and in women with late-onset disease (*p* = 0.0364), but not in the patients with early-onset preeclampsia (*p* = 0.331). The obtained results indicated a comparable level of endothelial injury in the two types of PE. The presence of a similar degree of endothelial injury in patients with EOP and LOP should create awareness among all clinicians about the possible fatal complications in both groups of patients with PE.

## 1. Introduction

Preeclampsia (PE) is one of the most dangerous and mysterious complications of pregnancy with extremely complicated but partially known pathophysiology. It is a disease originating from the placenta, which ultimately affects the maternal endothelium, leading to its injury and dysfunction [1]. Endothelial injury is probably responsible for the maternal complications of PE [2,3].

Researchers are intrigued by the differences in the clinical course of early- and late-onset preeclampsia (EOP and LOP), wherein patients with EOP exhibit a higher frequency of neurological, liver, and cardiorespiratory complications compared to those with LOP [2]. These differences may indicate the possible heterogeneity of PE with the two forms of the disease involving different pathophysiologies [3]. In addition, the endothelial injury level may also vary between these disease types.

Increased serum concentration of endothelial glycocalyx (EG) components, including hyaluronan (HA), constitutes one of the new markers of endothelial injury in PE [4,5]. A previous study showed that the degree of endothelial injury, expressed as the serum concentration of HA, was comparable in women with EOP and LOP [6].

Earlier, the degree of endothelial injury and dysfunction, was expressed by other markers, such as soluble cell adhesion molecules (sCAMs) [7,8,9]. Among them, serum level of soluble vascular cell adhesion molecule-1 (sVCAM-1) was higher in patients with PE than in healthy pregnant women [7,8,9]. In one of the studies it was significantly associated with patients with EOP compared to women with LOP [10].

Therefore, the present study aimed to assess the serum concentrations of HA and sVCAM-1 in patients with EOP and LOP and in healthy pregnant women.

## 2. Materials and Methods

The study was conducted among 60 women in their late second and third trimesters of a singleton pregnancy, including 20 patients with EOP, 20 patients with LOP, and 20 healthy women with normal pregnancy, who formed the control group. All the included women were hospitalized between 2015 and 2018 at the Division of Reproduction of the Poznan University of Medical Sciences. The women from the control group were of the same gestational age as the patients in the study groups.

The study was approved by the Ethic Committee of the Poznan University of Medical Sciences (no.1230/08) and written informed consent was obtained from all participants.

PE was characterized by hypertension (systolic blood pressure ≥140 mmHg or diastolic blood pressure ≥ 90 mmHg on two occasions) and proteinuria (≥300 mg/24 h), both of which occurred for the first time after 20 weeks of gestation [11].

PE was categorized as EOP when diagnosed at < 34 weeks of gestation and as LOP when diagnosed at ≥ 34 weeks of gestation.

Patients were recruited to the study groups immediately after the occurrence of the aforementioned signs (criteria) of PE and before commencing any medication.

Fetal growth restriction (FGR) was diagnosed when the ultrasound-estimated fetal weight was below the 10th percentile according to local growth charts and if the Doppler criteria for placental insufficiency were also met. The Doppler criteria included at least one of the following: increased (above the 95th percentile) uterine artery mean pulsatility index (PI), increased (above the 95th percentile) umbilical artery PI, decreased (below the 5th percentile) middle cerebral artery PI and decreased (below the 5th percentile) cerebroplacental ratio [12].

All the sonographic examinations, including the Doppler ultrasound, were conducted by experienced specialists using the E8 ultrasound system (GE Healthcare). The results of the latest Doppler examination, which was conducted at least 7 days before the delivery, were used for the final analysis.

To determine the concentration of HA and sVCAM-1 7,5 mL of venous blood was sampled from patients with PE on the day of diagnosis. After centrifugation of the blood samples (2000× *g*), the obtained serum was frozen at −20 °C until assessment. S-VCAM-1 and HA concentrations were determined by immuno-enzymatic tests (enzyme - linked immunosorbent assay (ELISA) kit procured from R&D Systems (Minneapolis, MN, USA) for sVCAM-1 and from EIAab (Wuhan, China) for HA). The assays were performed according to the manufacturer’s instructions. Plate reading was performed using an MRX reader (Dynex Technologies, Chantilly, VA, USA) at λ = 450 nm with corrections at 570 nm.

The exclusion criteria included the following: multiple pregnancy, fetal malformations, intrauterine infection, preterm premature rupture of membranes, sepsis, fever and pre-existing diabetes. Women exhibiting chronic hypertension were included in the study.

SigmaStat version 3.5 software (Systat Software, Inc., Point Richmond, CA, USA) was used for statistical analysis. The results were analyzed using one-way analysis of variance (ANOVA) with multiple comparison procedures and Student’s *t*-test for variables with parametric distributions. For variables with nonparametric distributions, Kruskal–Wallis ANOVA test by ranks with multiple comparison procedures and Mann–Whitney rank sum test were used. Spearman’s rank (r_s_) correlation test was used for determining the correlation between the serum concentration of sVCAM-1 and HA and the gestational age, blood pressure, proteinuria, body mass index (BMI) and age of patients with PE. The Chi-square test was used for assessing the parity distribution. *p* < 0.05 was considered statistically significant.

The authors conducted a post-hoc power analysis and found, with these number of included patients, 100% power to detect the differences between groups with PE and the control group for sVCAM-1 and 97% and 91% power for HA in EOP and LOP, respectively.

## 3. Results

The clinical characteristics of the patients are summarized in Table 1.

Statistical analysis: Mann Whitney test was used for comparison of the following data: age, gestational age at blood sampling, proteinuria; Chi-square test was used for comparison of the following data: parity (power 0.179), mode of delivery (power 0.988), percentage of FGR (power 0.615), percentage of fetal distress as an indication to caesarean section (power 0.923); Kruskal–Wallis ANOVA test by rank with multiple comparison was used for comparison of the following data: BMI of patients at onset of preeclampsia, gestational age at delivery, values of systolic and diastolic pressure; one way analysis of variance ANOVA with multiple comparison was used to compare newborn’s birth weight (power 0.974); FGR = Fetal growth restriction; PE = preeclampsia; NS = Not significant.

**Figure 1:**
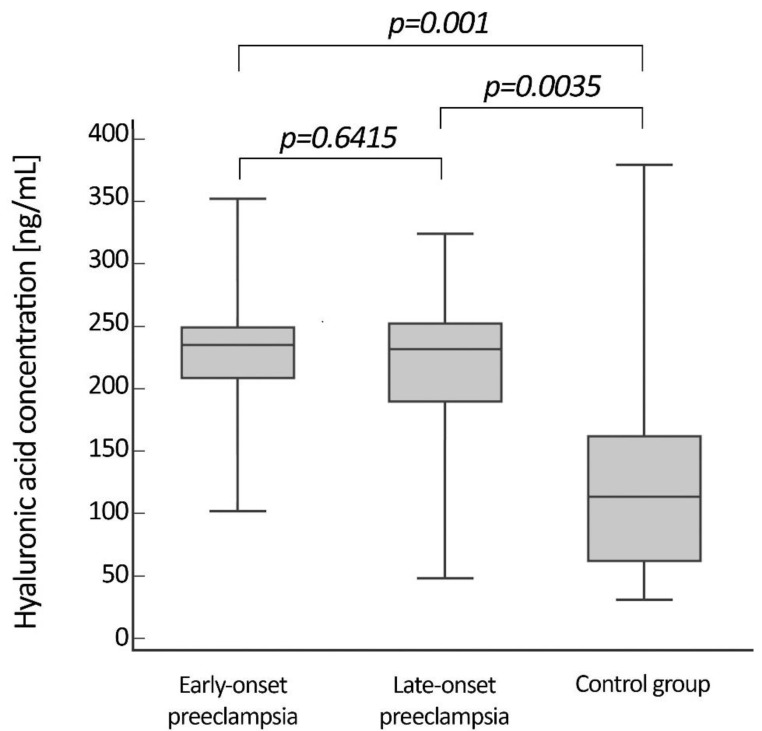
Serum concentrations of hyaluronan (HA) in patients with EOP, patients with LOP and in the normotensive women. Boxes describe interquartile range (25–75 percentile) and median, while whiskers describe min.–max. range. Statistical significance was assessed by Kruskal–Wallis ANOVA test by rank with multiple comparison.

The median concentrations of HA did not differ between patients with EOP and LOP. The values were 236.6 (101.1–351.9) ng/mL in patients with EOP and 234.7 (46.8–324.2) ng/mL in patients with LOP. However, the levels of HA in both patient groups were significantly higher than that of the control group (113.9 (30.9–379.8) ng/mL)). Figure 1 shows the serum level of HA in the two groups of patients and the control group.

**Figure 2:**
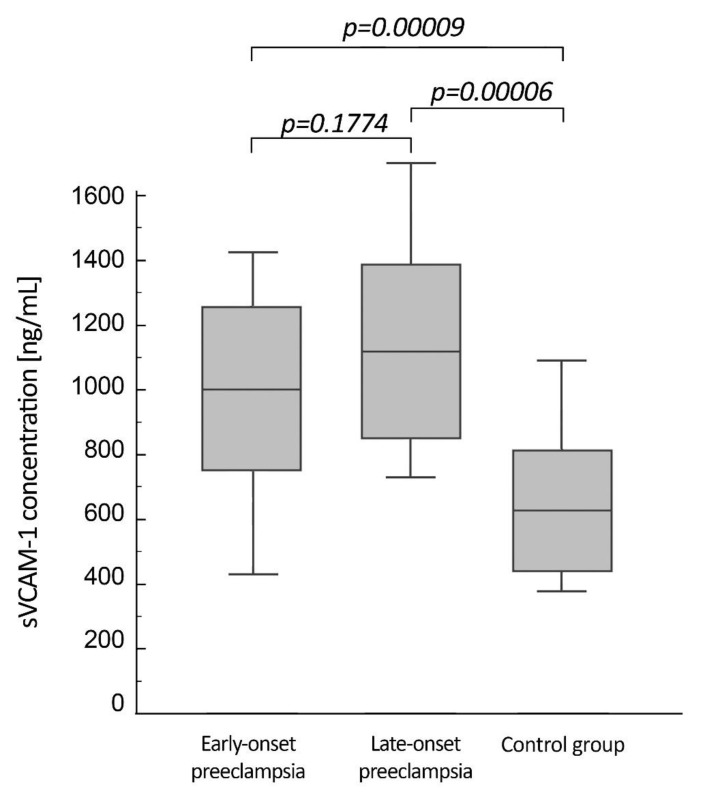
Serum concentrations of soluble vascular cell adhesion molecule-1 (sVCAM-1) in patients with EOP, patients with LOP and in the normotensive women. Boxes describe arithmetic mean ± SD, whiskers describe min.–max. range. Statistical significance assessed by one-way ANOVA with multiple comparison with a power of 0.999.

Similarly to HA, the mean serum level of sVCAM-1 did not differ statistically between patients with EOP (1003.8 ng/mL ± 253.7) and LOP (1119.7 ng/mL ± 277.5). Again, the concentrations of sVCAM-1 in both the patient groups were significantly higher than that of the healthy pregnant women (628.6 ng/mL ± 186.6). Figure 2 shows the serum level of sVCAM-1 in the two groups of patients and the control group.

The results of HA and sVCAM-1 also did not differ significantly (*p* = 0.607; *p* = 0.194) between patients with PE without FGR (237 ng/mL (46.8–324.2); 1103 ng/mL ± 260.4) and with FGR (226.6 ng/mL (101.1–351.9); 990.5 ng/mL ± 266), independent of gestational age at the onset of the disease.

**Figure 3:**
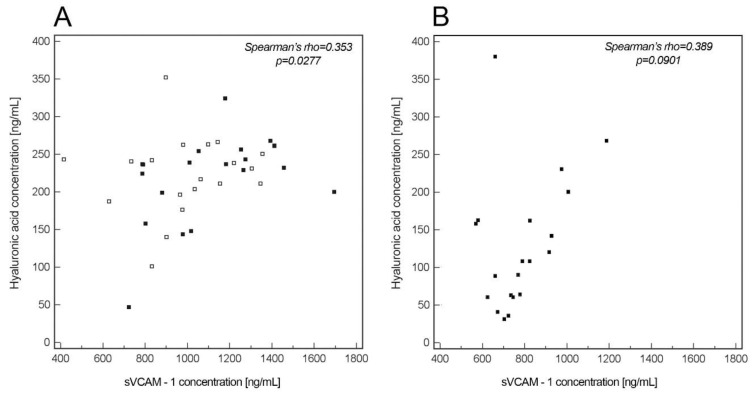
Correlation between serum concentrations of HA and sVCAM-1 concentrations in the entire group of patients with preeclampsia (PE) (**A**) and in the control group (**B**). Open squares correspond to cases of EOP (**A**), closed squares to LOP (**A**) and to the control group (**B**). Spearman ‘s rank test was used to assess the correlations.

A significant correlation was found between the levels of HA and sVCAM-1 in the entire group of patients with PE (*p* = 0.0277, r_s_ = 0.353, Figure 3A) as well as in patients with LOP (*p* = 0.0364, r_s_ = 0.494, Figure 3A), but not in the patients with EOP (*p* = 0.331, r_s_ = 0.221), nor in the control group (*p* = 0.0901, r_s_ = 0.389, Figure 3B).

In patients with EOP and LOP, respectively, no significant correlations were found between gestational age at the onset of PE (r_s_ for sVCAM-1 = 0.407, 0.0411; r_s_ for HA = −0.348, 0.378), age (r_s_ for sVCAM-1= 0.0415, 0.278; r_s_ for HA = 0.223, 0.0413), diastolic (r_s_ for sVCAM-1 = −0.192, 0.192; r_s_ for HA =−0.403, −0.121) and systolic blood pressure (r_s_ for sVCAM-1 =−0.298, −0.418; r_s_ for HA = −0.0983, 0.121), degree of proteinuria (r_s_ for sVCAM-1 = −0.21, 0.458; r_s_ for HA = 0.281, −0.272), and the levels of HA and sVCAM-1. A significant negative correlation was noted between BMI and the serum concentration of sVCAM-1 (r_s_ = −0.493), but only in the case of patients with LOP. Correlations between BMI and HA were not significant in patients with EOP (r_s_ = −0.201) and in patients with LOP (r_s_= −0.157).

The serum HA (*p* = 0.2490) and s-VCAM-1 (*p* = 0.2797) levels did not exhibit significant difference in the entire group of patients with PE with regard to the fetal gender. Similarly, no significant difference was found between the patients with LOP (*p* = 0.2519, *p* = 0.6369) and women in the control group (*p* = 0.4988, *p* = 0.4056). However, in the patients with EOP, the serum sVCAM-1 concentration was significantly higher in males than in females (*p* = 0.0419). Such a trend was not found for HA (*p* = 0.7018).

## 4. Discussion

One of the possible mechanisms of endothelial injury and dysfunction in PE is an increased production of soluble fms-like tyrosine kinase 1 (sFlt-1) by poorly perfused, hypoxic placental tissue [1,13]. SFlt-1 significantly contributes to endothelial injury by binding vascular endothelial growth factor (VEGF) [1,13]. One of the roles of VEGF is vascular and endothelial protection, which includes increasing the endothelial cell survival and stimulating the production of nitric oxide and prostacyclin [14]. Thus, in the presence of excess sFlt-1, the protective role of VEGF may be impaired.

Activation and dysregulation of maternal immune system constitutes the second most probable mechanism of endothelial damage and dysfunction in patients with PE [15,16]. The activation of immune system in patients with PE leads to a proinflammatory status, including intravascular inflammation [17]. This involves different types of cells including monocytes, neutrophils and others [16]. The former, among others, leads to an increased production of cytokines, whereas activation of neutrophils may more directly contribute do endothelial damage [16]. One mechanism leading to the activation of immune cells in women with PE may be the increased shedding of syncytiotrophoblast microparticles (STBM) into maternal circulation following the increased apoptosis of trophoblast cells in the placenta [16]. Interestingly, the primary dysfunction of immune system in mother may predispose to that by negatively affecting the proper trophoblast invasion in the first half of pregnancy [15].

In the present study, general endothelial dysfunction was found in patients with EOP and LOP. This was indicated by both the degree of EG degradation and the level of sCAMs, which is in line with the recent findings of increased EG degradation in patients with PE compared to normotensive pregnant women [4,5,6]. In 1997, Austgulen et al. found higher endothelial activation in patients with PE than in healthy pregnant women, indicated by the serum level of sVCAM-1 [18]. This finding was later confirmed by other studies [8,9,10], including the recent one by Docheva et al. [7].

Although the pathogenesis of EOP and LOP may be different, endothelial injury is involved in both of them. Various controversies concern the mechanism and the degree of endothelial injury in the two types of PE. The clinical observations and results of clinical studies suggest that the degree of endothelial injury and dysfunction may be higher in patients with EOP than in patients with LOP [2]. The higher incidence of neurological, cardiorespiratory and hematological complications in patients with EOP compared to patients with LOP may serve as evidence for these findings [2]. However, these have not been confirmed systematically in other clinical studies, in which the degree of endothelial injury was analyzed by determining the serum level of the EG degradation components, including HA and endocan, and the serum concentration of sVCAM-1 [5,6,9,19]. In the two studies conducted by Weissgerber et al. [5] and Dogan et al. [10], respectively, different results, concerning endothelial injury in patients with EOP and patients with LOP, were obtained. In the first study, the endothelial injury concerned only the level of syndecan-1 and not HA^,^ while in the second one, the level of sVCAM-1 was found to be significantly higher in patients with EOP than in patients with LOP. Thus, the results of the second study are in direct contrast with the present findings.

The comparable degree of endothelial injury in patients with EOP and LOP, found in the present study, indicates a complex pathomechanism of endothelial damage and dysfunction in PE. While in EOP it may be caused mainly by increased production of sFlt-1 by the placenta, there could be other different factors, except sFlt-1, responsible for endothelial injury in patients with LOP. These may comprise for example metabolic syndrome, including obesity which is often found in patients with LOP, as well as a higher gestational age at diagnosis of the late form of the disease. It is well known that there is pathophysiological relation between metabolic syndrome and obesity and the increased vascular wall inflammation [20].

As the in-depth assessment of endothelial dysfunction by analyzing the degree of EG degradation has not yet been attempted, the authors of this study aimed to find out if this new assessment technique is consistent with other, well-established ways of examining this phenomenon.

The levels of sVCAM-1 in patients with EOP, and LOP, and normotensive pregnant women turned out to be very similar to the levels of HA in all the three groups. The results of the present study provide important evidence for the earlier findings on the comparable degree of endothelial injury and dysfunction in EOP and LOP. Moreover, the obtained results show a complex mechanism of endothelial dysfunction in PE. The increased shedding of EG components into the blood indicates significant degradation of the endothelial surface layer in PE. Concerning the critical role of EG in protecting endothelium, including maintenance of tissue integrity, prevention of leukocytes and platelet adhesion, and antithrombotic activity, it is understandable that the following consequence of EG degradation leads to further activation of endothelium with the participation of the above phenomena [21,22,23]. This also includes the increased expression of sCAMs, as VCAM-1. Activation of these mechanisms leads to inflammation caused by increased migration of leukocytes into the subendothelial space [24]. This process along with the prothrombotic activity resulting from endothelial injury and activation may further exacerbate the underlying pathology of PE with all known clinical consequences.

Interestingly, despite very similar mean results of HA and sVCAM-1 in all three patient groups, there was greater overlap in variance for HA compared to sVCAM-1 between the preeclamptic groups and the control group, which was caused in fact by only two patients from the control group exhibiting high HA level. Unfortunately, the authors did not find any possible clinical cause for this phenomenon and it definitely requires further investigations in future. The aforementioned differences may possibly indicate the better usefulness of sVCAM-1 than HA in distinguishing between patients with and without endothelial injury.

When analyzing the potential effect of the fetal gender on the degree of endothelial injury in patients with PE, the authors did not find any distinct trend. It is in contrast to some previous findings on possible increased risk of endothelial damage associated with female fetuses [25,26]. Interestingly, Zhou et al. [25] found the increased vulnerability of female fetal endothelial cells in pregnancies complicated by PE, whereas Andersen et al. [26] found a higher concentration of sFlt-1 in patients with PE and female fetuses compared to patients with PE and male fetuses.

Although PE is still considered to be heterogenous, owing to the complex pathology of pregnancy, the obtained results indicate that the crucial and ultimate pathophysiological phenomenon of PE is very similar in all patients with this disease. These findings should have implications in the management of these patients, with the awareness that fatal complications could occur in both patients with EOP and patients with LOP. Some data show that severe maternal complications occur more often in patients with EOP than in patients with LOP. This may be because patients with LOP deliver soon after the diagnosis of PE, which saves them from these complications.

Another implication for the future could be that increased information on the pathophysiology of the disease would aid in the development of new treatment strategies, including those associated with the restoration of endothelial injury. So far, there has been a great progress in the prophylaxis of PE, but not in the treatment of the disease.

A limitation of the study is the small number of patients included in the study groups. The strength of the study might be that this is the first comparison study on both well-established markers of endothelial activation and the possible new ones, which directly reflect endothelial damage in PE.

## 5. Conclusions

The obtained results indicated a comparable level of endothelial injury in the two types of PE. The similar degree of endothelial injury in patients with EOP and LOP should create awareness among all clinicians about the possible fatal complications in both groups of patients with PE.

## Figures and Tables

**Table 1:** Clinical characteristics of women with early-onset preeclampsia (EOP) and late-onset preeclampsia (LOP) and with normal pregnancy.

	Early-Onset PE (EOP) (n = 20)	Late-Onset PE (LOP) (n = 20)	Normal Pregnancy (NP) (n = 20)	P EOP vs. LOP	P EOP vs. NP	P LOP vs. NP
Age (years) median (range)	28 (25–41)	31 (19–40)	32 (27–36)	0.103	**0.029**	0.314
Parity n (%) 0 ≥1	8 (40) 12 (60)	13 (65) 7 (35)	11 (55) 9 (45)	0.2802		
BMI at onset of preeclampsia median (range)	26.4 (21.8–40.6)	34.3 (22.7–53.2)	25 (22–29)	**0.0168**	0.2420	**0.0009**
Gestational age at blood sampling (weeks) median (range)	28 (24–32)	33 (34–38)	31 (23–37)	**0.0009**	**0.0248**	0.0511
Gestational age at delivery (weeks) median (range)	32 (26–37)	37 (34–39)	39 (37- 41)	**0.0005**	**0.0007**	**0.01**
Mode of delivery n (%) Vaginal delivery Caesarean section	0 20 (100)	4 (20) 16 (80)	8 (60) 12 (40)	**0.0209 with Yates correction**		
FGR n (%)	12 (60%)	4 (20%)		**0.0098**		
Fetal distress as an indication to caesarean section n (%)	13 (65%)	2 (10%)		**0.0003**		
Mean systolic pressure (mmHg) median (range)	150 (130–180)	150 (130–180)	110 (100–120)	0.3107	**0.0009**	**0.0009**
Mean diastolic pressure (mmHg) median (range)	90 (90–110)	100 (85–130)	70 (60–80)	0.3980	**0.0009**	**0.0004**
Proteinuria (g/24 h) median (range)	3.78 (0.3–10.5)	3.86 (0.4–12.9)		0.6917		
Newborn’s birth weight (g) mean	1319 ± 568	2636 ± 683	3437 ± 554	**0.0008**	**0.0009**	**0.0006**
Fetal gender n (%) Boy Girl	9 (45%) 11 (55%)	10 (50%) 10 (50%)	10 (50%) 10 (50%)	0.9354		
